# U94 alters *FN1 *and *ANGPTL4 *gene expression and inhibits tumorigenesis of prostate cancer cell line PC3

**DOI:** 10.1186/1475-2867-5-19

**Published:** 2005-06-22

**Authors:** Ekwere T Ifon, Alan LY Pang, Warren Johnson, Kathleen Cashman, Sharon Zimmerman, Sumitra Muralidhar, Wai-Yee Chan, John Casey, Leonard Jason Rosenthal

**Affiliations:** 1 Department of Microbiology and Immunology, Georgetown University Medical Center, 3900 Reservoir Road, NW, Washington, D.C. 20057, USA; 2 Laboratory of Clinical Genomics, NICHD, National Institutes of Health, 9000 Rockville Pike, Bethesda, MD 20892, USA; 3 Department of Pediatrics, Georgetown University Medical Center, 3800 Reservoir Road, NW, Washington, D.C. 20057, USA; 4 Department of Cell Biology, Georgetown University Medical Center, 3800 Reservoir Road, NW, Washington, D.C. 20057,; 5 Department of Biochemistry & Molecular Biology, Georgetown University Medical Center, 3800 Reservoir Road, NW, Washington, D.C. 20057, USA;

## Abstract

**Background:**

Insensitivity of advanced-stage prostate cancer to androgen ablation therapy is a serious problem in clinical practice because it is associated with aggressive progression and poor prognosis. Targeted therapeutic drug discovery efforts are thwarted by lack of adequate knowledge of gene(s) associated with prostate tumorigenesis. Therefore there is the need for studies to provide leads to targeted intervention measures. Here we propose that stable expression of *U94*, a tumor suppressor gene encoded by human herpesvirus 6A (HHV-6A), could alter gene expression and thereby inhibit the tumorigenicity of PC3 cell line. Microarray gene expression profiling on *U94 *recombinant PC3 cell line could reveal genes that would elucidate prostate cancer biology, and hopefully identify potential therapeutic targets.

**Results:**

We have shown that stable expression of *U94 *gene in PC3 cell line inhibited its focus formation in culture, and tumorigenesis in nude mice. Moreover gene expression profiling revealed dramatic upregulation of *FN 1 *(fibronectin, 91 ± 16-fold), and profound downregulation of *ANGPTL 4 *(angiopoietin-like-4, 20 ± 4-fold) in *U94 *recombinant PC3 cell line. Quantitative real-time polymerase chain reaction (QRT-PCR) analysis showed that the pattern of expression of *FN 1 *and *ANGPTL 4 *mRNA were consistent with the microarray data. Based on previous reports, the findings in this study implicate upregulation of *FN 1 *and downregulation of *ANGPTL 4 *in the anti tumor activity of U94. Genes with cancer inhibitory activities that were also upregulated include *SERPINE 2 *(serine/cysteine protease inhibitor 2, 7 ± 1-fold increase) and *ADAMTS 1 *(a disintegrin-like and metalloprotease with thrombospondin type 1 motif, 7 ± 2-fold increase). Additionally, *SPUVE 23 *(serine protease 23) that is pro-tumorigenic was significantly downregulated (10 ± 1-fold).

**Conclusion:**

The dramatic upregulation of *FN 1 *and downregulation of *ANGPTL 4 *genes in PC3 cell line stably expressing U94 implicate up-regulation of *FN 1 *and downregulation of *ANGPTL 4 *in anti tumor activity of U94. Further studies are necessary to determine functional roles of differentially expressed genes in *U94 *recombinant PC3 cell line, and hopefully provide leads to potential therapeutic targets in prostate cancer.

## Background

Prostate cancer is the most common form of malignancy in US males. An estimated 29,900 fatalities out of 230,110 new cases are expected in the year 2004 [1]. Androgen ablation is currently the mainstay in prostate cancer therapy, but its efficiency is marred by the relapse of some advanced-stage prostate cancer cells into an androgen refractory state [2,3]. Advanced-stage prostate cancer progression is usually aggressive and correlates with poor prognosis [2,4,5]. Hence, insensitivity to androgen ablation by advanced-stage prostate cancer invariably constitutes a major problem in clinical therapy. Therefore there is an urgent need for the development of targeted therapeutic strategies in advanced-stage prostate cancer.

Knowledge of the genes that are associated with prostate cancer is important for designing an effective therapeutic strategy. However, present knowledge of the molecular biology of prostate cancer is inadequate to define etiologic genes [5-7]. Consequently, current therapeutic strategies in prostate cancer are inefficient [8-20], and an effective targeted therapy remains elusive. This situation prompted our laboratory to embark on studies to provide alternative leads for the development of efficacious and targeted anti prostate cancer agent(s). In our approach, we investigated the anti tumor activity of U94 protein (U94) in prostate cancer cell line, PC3.

*U94 *is a 1473 bp gene located in the HD12 fragment of human herpesvirus 6A (HHV-6A), strain U1102 [21]. *U94 *encodes a 490 amino acid protein that is not found in other herpesviruses [21,22], and U94 is expressed at very low levels [23,24]. Recent reports suggest that *U94 *is a latency gene, and modulates viral DNA replication [23-26]. Moreover, structural homology of U94 to Rep 78/68 from adeno-associated virus type 2 (AAV-2) [21,27] suggests that there might be functional similarities between these proteins. Strong evidence in support of functional similarities between U94 and Rep 78/68 is the observation that U94 complemented the replication of an AAV-2 mutant that was deficient in Rep 78/68 [28]. Additionally, recent reports show that U94 also inhibits gene transcription [29], which is a biological function of its homologue Rep78/68. However, U94 may affect gene transcription differently than Rep 78/68, because U94 activates human immunodeficiency virus 1 (HIV-1) long terminal repeat (LTR) promoter in fibroblast cell lines [28] and inhibits HIV-1 LTR in T-cell lines [29], whereas Rep 78/68 inhibits HIV-1 LTR promoter in both fibroblast cell lines and T-cell lines [28].

Previous studies demonstrated that U94 suppressed transformation by oncogenes [22,29]. Data from these studies showed that an NIH 3T3 cell line stably expressing *U94 *gene suppressed transformation by the oncogene *H-ras*, when compared to the parental NIH 3T3 cell line treated under similar conditions [29]. We were motivated by the findings in previous studies to determine the anti tumor potential of U94 in the human prostate tumor cell line PC3.

**Figure 2:**
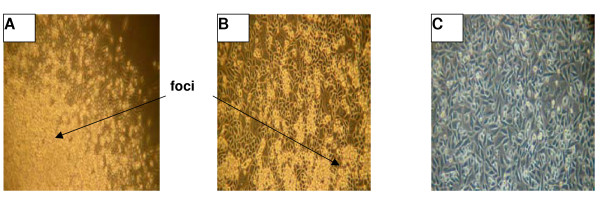
**Inhibition of focus formation by PC3 cell line stably expressing U94 protein. **PC3 cell line transfected with plasmid containing *U94 *DNA was used for studies. The controls were PC3 cell line transfected with vector cassette or parental PC3 cell line. 1 × 10^6 ^cells/ 75 cm^2 ^culture flask was grown to confluence, and focus formation was examined 10 days after. Panel A: PC3 cells (negative control); Panel B: PC3 cells transfected with vector cassette (vector control); Panel C: PC3 cells stably expressing U94 protein (test). Control cells formed foci (Panel A and Panel B) consisting of rounded refractive cells piling on top of each other. Notice that the expression of U94 protein (Panel C) inhibited focus formation. (Magnification: 20X on Olympus CK2 microscope, Olympus Optical Co. Ltd. Japan).

**Figure 3:**
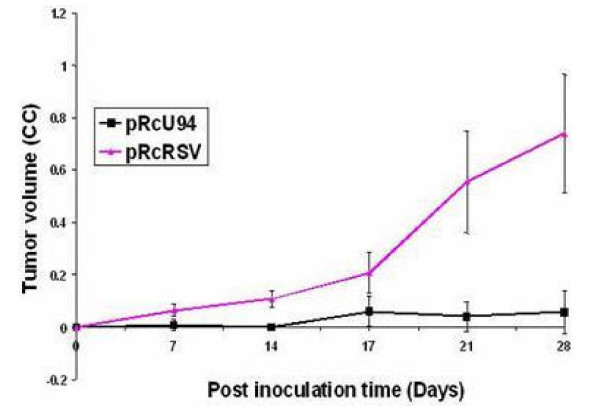
**Tumorigenicity of U94 recombinant PC3 cell line in nude mice. **Stable G418 resistant cell lines were generated by transfection of PC3 cells with either pRc-RSV (vector control) or pRc-U94 (test). Confluent cells (5 × 106 cells/100 μl), in culture medium without antibiotics or serum were inoculated behind the neck into athymic nude mice (Ncr nu/nu), and monitored for tumor production. Tumor size was measured on days 7, 14, 17, 21 and 28 post inoculation. Data from two animal experiments (experiment 1: n = 3 per group; and experiment 2, n = 4 per group) were pooled. The average tumor volume in cubic centimeters was plotted against time in days. The error bars represent standard deviation. Notice the significant reduction in tumor size in animals inoculated with U94 recombinant PC3 cell line. A repeated measures analysis of variance demonstrated a significant difference (P < 0.05) in tumor volume between test and control animals. Additionally, paired Student's t-test showed a significant difference in average tumor size of test animals in comparison to control animals.

**Figure 4:**
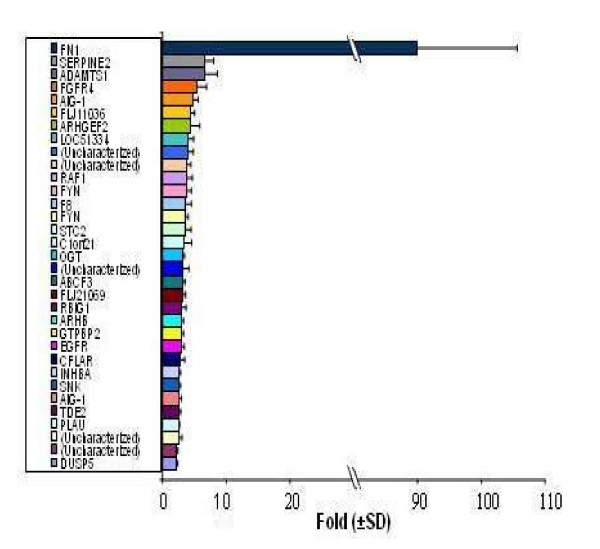
**Upregulated genes in PC3 cell line stably expressing U94**. Two clones of G418 resistant PC3 cell lines transfected with plasmid pBk-U94 (tests 1 and 2) and a clone transfected with plasmid pBK-CMV (reference) were used for cDNA microarray studies. Each experiment was performed in triplicate and the results are mean ± SD. Notice the dramatic upregulation of *FN 1 *(91 ± 16-fold). A subset of other genes was also upregulated, but genes of interest in this study (> 6-fold change) include: *SERPINE 2 *(7 ± 1-fold); and *ADAMTS 1 *(7 ± 2-fold).

**Figure 5:**
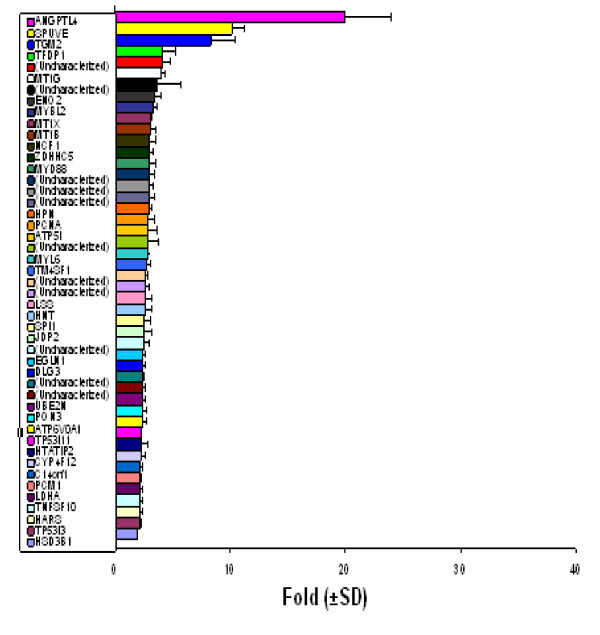
**Downregulated genes in PC3 cell line stably expressing U94. **Data presented here were generated as reported in Figure 4. Notice the pronounced down-regulation of angiogenic gene, ANGPTL4 (20 ± 4-fold). Additional genes that were significantly downregulated, and with a fold change > 6, include: SPUVE 23 (10 ± 1-fold); TGM 2 (8 ± 2-fold).

In this paper we report that the expression of U94 protein in PC3 cells inhibited foci formation (Figure 2; Table 1), and the tumorigenicity of recombinant PC3 cell line in athymic nude mice (Figure 3). Moreover, gene expression analyses (Figures 4 and 5), and QRT-PCR (Table 2) revealed dramatic upregulation of *FN 1 *(~91-fold) and profound downregulation of *ANGPTL 4 *(~20-fold) in 2 separate recombinant PC3 cell lines stably expressing U94. Our study also demonstrated the differential expression pattern of several other genes in the presence of U94. This is the first study to report the inhibitory potential of U94 on the tumorigenicity of advanced-stage prostate cancer cell line PC3.

**Table 1:** Inhibition of focus formation by prostate cancer cell line PC3 expressing U94 protein.

**Cell line**	**No. of foci/75 cm**^2^**flask**
Parental PC3 cell line	80
Vector transfected PC3 cell line	69
PC3 cell line stably expressing U94	2

Clonal PC3 cell lines, transfected with plasmid containing U94 gene and geneticin (G418) resistant cassette, were used for studies. PC3 cell line transfected with vector cassette or parental PC3 cell line served as controls. G418 resistant clones were sub-cultured and grown to confluence. Focus formation was detected as dense foci of actively growing and refractive cells. The experiment was performed in duplicate, and the average number of foci was reported. Notice that stable expression of U94 drastically inhibited focus formation.

**Table 2:** Fold changes of differentially expressed genes in PC3 cell line stablyexpressing U94.

**PC3/U94 Clones**	***FNI***	***SERPINE2***	***ADAMTS1***	***ANGPTL4***	***SPUVE23***
			**Microarray**		
1 & 2	91 ± 16	7 ± 1	7 ± 2	-20 ± 4	-10 ± 1
			**QRT-PCR**		
1	183 ± 27	3 ± 1	4 ± 1	-76 ± 13	-6 ± 1
2	467 ± 33	7	10 ± 1	-67 ± 5	-9

Differential expression of genes was determined by microarray, and QRT-PCR was used to confirm microarray data. Microarray data were computed from array analyses presented in Figures 4 and 5. For QRT-PCR, a subculture of PC3 cell lines used for microarray was used for studies: 2 clones of *U94 *transfected PC3 cells and vector transfected PC3 cells. The protocol for total RNA extraction was same as for microarray studies, except that the RNA extracts were treated with DNase 1. QRT-PCR was performed in triplicate, using SYBR^® ^Green I chemistry, on 7900 HTS Sequence Detection System (Applied Biosystems, Foster City, CA) according to manufacturer's instructions. The results are presented as average of 3 experiments ± SD. Notice that Microarray data and QRT-PCR data show similar trends; moreover the trend of QRT-PCR results was reproducible in 2 clones of *U94 *recombinant PC3 cell lines.

## Results

Previously, we have demonstrated that U94 inhibited gene transcription and also transformation by oncogenes [22,29]. Several reports have implicated the malfunction of transcription regulatory factors [30-38] as well as the activities of oncogenes [39-48] as etiologic factors in prostate tumorigenesis. Hence we wanted to determine whether U94 could exert inhibitory activity on the tumorigenesis of PC3 cell line.

### Expression and intracellular localization of U94 protein

**Figure 1:**
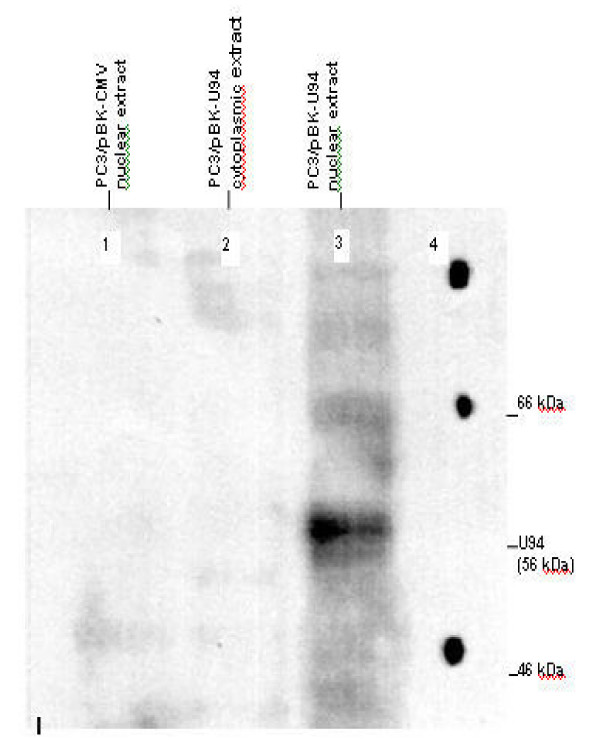
**Immunoblot of U94 protein. **Nuclear and cytoplasmic protein was extracted from confluent PC3 cell line, PC3 cell line transfected with vector cassette (controls) or PC3 cell line transfected with plasmid containing U94 DNA (test). 100 μg sample protein was loaded per lane and separated by SDS-PAGE through a 10% Tris-glycine gel (Novex; Invitrogen, Gaithersburg, MD). The results showed the 56 kDa U94 protein in the nuclear extract of U94 recombinant cells. There was no immunoreactivity in all cytoplasmic extracts, and nuclear extracts of control cells. U94 protein was detected using the polyclonal anti-U94 antibody, AB679 (Amersham, England). The positions of 46 kDa and 66 kDa markers, and the U94 protein are indicated on the right.

First, we wanted to determine whether U94 could be expressed in PC3 cell line. We transfected PC3 cell line with plasmid pBKU94, which contained *U94 *DNA insert and a selectable geneticin (G418)-resistant vector cassette. pBKCMV vector transfected PC3 cells served as control. Immunoblot analyses, using the U94 polyclonal antibody AB679 as depicted in Figure 1, showed that U94 (56 kDa) protein was expressed in the nuclear fraction (lane 3) and not the cytoplasmic fraction (lane 2) of stably transfected PC3 cell line. No immunoreactivity was detected in the nuclear fraction of vector transfected PC3 cell line (lane 1). Figure 1 lane 4 shows the high molecular weight Rainbow marker.

### Inhibition of focus formation by U94 protein expression

In order to monitor the effect of U94 on tumor formation, we investigated focus formation by PC3 cell line as an index of a neoplastic phenotype. Focus formation was observed as dense foci of intensive cell growth in culture, consisting of refractive cells that rounded up and piled on top of each other [49]. Three PC3 cell lines were used in this study: *U94 *transfected, vector cassette transfected, and parental PC3 cell line. For each cell line, 1 × 10^6 ^cells/ 60 mm culture dish was seeded and grown to confluence. Focus formation was examined 10 days post confluence. The result of this study (Table 1) showed a drastic reduction in focus formation by PC3 cells expressing U94: the number of foci were reduced ~35-fold and 40-fold in comparison with the control vector transfected and parental PC3 cell lines, respectively. Figure 2 shows large and widespread foci in the culture of control vector transfected and parental PC3 cell lines. The culture of recombinant PC3 cell line expressing U94 protein showed only few foci, grossly reduced in size. Our findings suggest that U94 may exhibit anti tumor activity *in vitro*.

### Expression of U94 inhibits tumorigenicity of PC3 cell line in athymic nude mice

In order to determine whether U94 inhibits the tumorigenicity of PC3 cell line *in vivo*, we inoculated 5 × 10^6 ^PC3 cells (*U94 *transfected cells as test, or vector cassette transfected cells as control) subcutaneously behind the neck, into athymic nude mice. Animals were examined for tumor formation on days 7, 14, 17, 21, and 28 after inoculation. Our result showed that tumor formation was inhibited in mice that were inoculated with PC3 cell line stably expressing U94 protein (Figure 3). The control animals that were inoculated with PC3 cell line transfected with vector cassette developed tumors, and tumor size increased progressively with time as shown in Figure 3. Statistical analysis, using a repeated measures analysis of variance (ANOVA), demonstrated that tumor volume in test and control animals were significantly different (P < 0.05). A comparison of tumor volume between test and control mice, using Paired Student's t-test to supplement ANOVA, further showed that the average tumor volume of test and control animals were significantly different (P < 0.05) on each day tumor volumes were determined. These findings demonstrate that U94 significantly (P < 0.05) inhibited the tumorigenicity of PC3 cell line in athymic nude mice, and corroborate our data (Figure 2) from focus formation assay.

### Microarray gene expression profiling in PC3 cell line stably expressing U94

We performed microarray gene expression profiling on recombinant PC3 cell line stably expressing U94 to determine whether U94 affected expression of genes involved in tumorigenesis. We used two clones of *U94 *recombinant PC3 cell lines as test samples, and PC3 cell line transfected with plasmid vector as our reference. The effect of U94 on gene expression was analyzed using two-color comparative fluorescence assays on glass slide microarrays containing ~6,000 cancer related genes. Our data demonstrated the differential expression of 78 genes: 31 genes were upregulated (Figure 4) while 47 genes were downregulated (Figure 5). These results show the mean values for two clones of *U94 *recombinant PC3 cell lines. Notably, the microarray results revealed dramatic upregulation of *FN 1 *(91 ± 16-fold), and profound downregulation of *ANGPTL 4 *(20 ± 4-fold) in PC3 cell lines stably expressing U94 protein. Although a majority of the differentially expressed genes showed a 2–3 fold change in expression level in the presence of U94, we decided to consider for further studies only genes with ≥ 6-fold change. The microarray data is deposited in the Gene Expression Omnibus of the NCBI, and is available at the NCBI web site .

### Quantitative real-time PCR

We performed QRT-PCR to confirm the microarray data. In one clone of *U94 *recombinant PC3 cell line, the QRT-PCR data (Table 2) showed that the changes in expression levels of *FN 1 *and *ANGPTL 4 *mRNA were 183 ± 27-fold increase and 76 ± 13-fold decrease, respectively. In the second clone of *U94 *recombinant PC3 cell line, the corresponding changes in expression levels were 467 ± 32-fold increase and 67 ± 5-fold decrease, respectively. The observed differences in the fold changes between the microarray and QRT-PCR data may be due, at least in part, to differences in detection sensitivity of the two techniques, as well as the subtle differences in experimental conditions and physiological conditions in the microenvironment of the cells in culture. Nevertheless, the trend observed from QRT-PCR data was consistent with the trend from microarray data. Additionally, the results from both techniques showed elevation of *SERPINE 2 *and *ADAMTS 1 *expressions (Table 2 and Figure 4), and downregulation of *SPUVE 23 *(Table 2 and Figure 5). TGM-2 (transglutaminase 2) showed 8 ± 2-fold decrease by microarray, but we did not perform QRT-PCR.

## Discussion

In the present study, we have demonstrated for the first time that U94 protein inhibited focus formation and tumorigenicity of the prostate cancer cell line PC3. This study is particularly interesting because PC3 cell line is a derivative of advanced-stage prostate cancer metastasis to bone and is insensitive to androgen ablation therapy. Insensitivity to androgen ablation therapy is associated with aggressive progression of the cancer, and ultimately fatal in less than 24 months [2,6]. Therefore the anti tumor activity of U94 in PC3 cell line is novel and interesting, and may have a translational application.

The impetus for our study on anti tumor activity of U94 in PC3 cell line was given by previous findings [22,29] that U94 suppressed transformation by oncogenes. Apparently, U94 shares this functional activity with its homologue Rep 78/68 of AAV-2 [24]. However, the mechanism(s) of transformation suppressor activity is not understood. A previous report showed that U94 lost its activity when translation termination linkers were inserted at codons 25, 125 and 245 of its nucleotide sequence [29]. This finding implicates U94 protein expression in anti tumor activity in recombinant PC3 cell line. Therefore we performed immunoblot analysis and demonstrated that U94 protein (56 kDa) was expressed, and localized to the nucleus (Figure 1, lane 3) in PC3 cell line. Nuclear localization of U94 protein suggests that U94 might exert activity, probably on gene expression, in the nucleus of PC3 cell line. This view is in consonance with previous findings [22,29] that U94 inhibited gene expression. A previous study [29] showed that U94 suppressed the P97 promoter, which controls the expression of the *E6 *and *E7 *transforming genes of human papillomavirus 16 (HPV-6). Therefore we suspect that the tumor suppressor activity of U94 in our study was exerted by inhibition of gene expression. It is interesting to note that the expression of U94 protein does not affect the growth pattern of NIH 3T3 cell line [29]. This observation is supported by another report [25] that lymphoid cells stably expressing U94 had the same morphology and growth characteristics as parental cell line. Thus previous findings suggest that U94 is not toxic to cells, and we speculate that the same would be true for the PC3 cell line.

In the current investigation, we examined the effect of stable expression of U94 protein on focus formation, a malignant phenotype, by recombinant PC3 cell line. Focus formation by PC3 cell line stably expressing U94 was inhibited drastically (~30- to 40-fold) in comparison to that of control parental and vector transfected cell lines (Table 1). As shown in Figure 2, widespread and large foci were formed by control PC3 cell lines in contrast to background foci formed by *U94 *recombinants. It is possible that the few foci observed in the background originated from spontaneous transformation and/ or leakage during clonal selection. Furthermore, our studies demonstrated that the anti cancer activity of U94 was sustainable *in vivo *as tumor development was significantly (P < 0.05) inhibited in mice that were treated with U94 recombinant PC3 cell line (Figure 3). Previous studies linked prostate malignancy to the activities of oncogenes [39-48]. Therefore the inhibition of focus formation and tumorigenicity in our study supports the hypothesis that U94 probably inhibited oncogenic activities in prostate cancer cell line PC3, and thereby exhibited anti cancer activity. Our microarray data identified *FN 1 *that was dramatically elevated (~91-fold) (Figure 4) and *ANGPTL 4 *that was profoundly reduced (~20-fold) (Figure 5) as genes of interest in this study. Up-regulation of *FN 1 *in the current study was actually unexpected because previous reports [22,29] suggested that U94 inhibited gene expression. In contrast, our findings suggested that U94 actually altered gene expression in PC3 cell line positively or negatively. Although it is not known how U94 mediated gene expression, our data is interesting because *ANGPTL 4 *is pro-angiogenic [50], and reduced expression could negatively impact tumorigenesis. Additionally, previous reports [51-54] implicated elevated *FN 1 *and/ or its derivatives in paradigms of tumor inhibition.

Fibronectin (FN) is a major component of extracellular matrix (ECM), where it is assembled as insoluble polymers, and is present in the blood as a soluble dimer [55]. Fibronectin 1 (FN 1) is a homologue of FN, and contains a self-assembly domain, which induces FN 1-FN 1 polymerization [56-58]. Therefore the terms FN 1 and FN are used interchangeably in regard to polymerization in this report.

FN 1-FN 1 interaction is reported [59,60] to induce conformational changes that increase the binding ability of FN 1 to receptor(s). We speculate that the tremendous upregulation of *FN 1 *transcription in the presence of U94 led to elevated translation and secretion of protein product in PC3 cells. The increased level of FN 1 protein in turn accelerated FN 1-FN 1 polymerization [56-58]. We suspect that polymeric FN 1 binding to PC3 cell surface mitigated malignant signaling [61]. The potential anti-malignancy activity of FN 1 is evident from a recent report [62] that exogenous FN 1 could reverse transformed phenotype. Hence, FN 1 interaction with PC3 cell surface might have contributed, at least in part, to inhibition of focus formation *in vitro *(Figure 2) and tumorigenesis *in vivo *(Figure 3).

Strong support for *in vivo *anti tumor activity of polymeric FN 1 is given by a previous report [53] demonstrating that systemic administration of polymeric FN 1 exhibited anti tumor activity in mice bearing various types of tumors. Further support is provided by a recent report [59] showing that anastellin, a component of FN 1 that is capable of inducing FN 1-FN 1 polymerization, displays anti angiogenic and anti metastastic properties *in vivo*. Additionally, other workers [52] have demonstrated that peptides of FN 1 exert anti tumor activity *in vivo*. These findings provide a rational explanation for the observation in this study that elevated FN 1 expression in *U94 *recombinant PC3 cell line is associated with anti tumor activity. Taken together, previous reports [52,53,59] lend credence to our view that binding of polymeric FN 1 to PC3 cells surface induces the inhibition of focus formation in culture and inhibition of tumorigenicity in mice. However, in addition to *FN 1*, our results also implicated *ANGPTL 4 *in the anti tumor activity of U94 in PC3 cell line.

*ANGPTL 4 *was shown in a chicken chorioallantoic membrane assay to induce a strong pro-angiogenic response, independent of *VEGF *gene [50]. Since angiogenesis is implicated in vascular development, and neovascularization is the hallmark of tumor progression [63,64], an inhibitor of angiogenesis could greatly impact tumor therapy. In the current study we have demonstrated that *ANGPTL 4 *was profoundly inhibited (downregulated about 20-fold) in *U94 *recombinant PC3 cell line. It is therefore expected that downregulation of *ANGPTL 4 *would exert a negative effect on vascular development, and thereby inhibit PC3 cell line tumorigenicity *in vivo*. Although it is not clear how U94 mediates the expression of *ANGPTL 4*, recent reports [65-67] show that angiogenesis is regulated by ECM signals. Interestingly, other reports [53,59] suggest that the anti-angiogenic property of polymeric FN 1 is mediated by induction of ECM signals. Therefore, it appears that there may be a casual or causal relationship between anti tumor activity of polymeric FN 1 and the inhibition of *ANGPTL 4 *in *U94 *recombinant PC3 cell line. Since *ANGPTL 4 *supports vascular development [50], we speculate that *ANGPTL 4 *did not mediate the inhibition of focus formation by PC3 cell line in this study.

In addition to *FN 1 *and *ANGPTL 4*, we also chose for further studies a subset of other genes that expressed differentially > 6-fold. Genes in this category included *SERPINE 2 *(elevated ~7-fold), *ADAMTS 1 *(upregulated ~7-fold) and *SPUVE 23 *(downregulated ~10-fold). *SERPINE 2 *encodes a serine proteinase inhibitor, and was recently implicated in anti cancer activity [68]. *ADAMTS 1 *is an active metalloproteinase associated with ECM [69]. It is essential for normal growth [70], but also displays anti-angiogenic activity [71]. In consonance with previous reports [68,71], data from the current study suggest that *SERPINE 2 *and *ADAMST 1 *probably exerted anti tumor activity. Previous studies [72,73] showed that the expression of *SPUVE 23*, a serine protease, is associated with increased malignant potential. Therefore we propose that downregulation of *SPUVE 23 *in *U94 *recombinant PC3 cell line is tantamount to anti tumor activity.

In conclusion, the findings in this study have suggested that U94 exhibits anti tumor potential in PC3 cell line. The dramatic elevation of *FN 1 *expression and reduction of *ANGPTL 4 *expression in U94 recombinant PC3 cell line can be interpreted as evidence of the mechanism of U94 anti tumor activity. Therefore data from our study seem to support the anti tumor hypothesis of FN 1 previously reported by other workers [51,52,54,55,74]. Moreover, this report identifies *ANGPTL 4 *and *SPUVE 23 *as potential therapeutic targets in prostate tumorigenesis. Hopefully, further studies on the microarray data reported herein might elucidate the complex genetic alterations that underlie advanced-stage prostate tumorigenesis, and thereby provide leads for defining targeted therapeutic strategies for advanced-stage prostate cancer.

## Materials and methods

### Cells and transfection

PC3 cell line was purchased from American Type Culture Collection (ATCC, Manassas, VA, USA) and plasmid *U94 *DNA was prepared as previously described [22,29]). All cells were cultured in HAM's F12 medium (Cell gro/Mediatech, VA, USA) supplemented with 2 mM glutamine, 100 U of penicillin-streptomycin per ml (Invitrogen, Gaithersburg, MD, USA), and 10% Fetal Bovine Serum (FBS, HyClone, Logan, UT, USA), at 37°C and 5% CO_2_. Plasmid *U94 *DNA was cloned into the HindIII site of pRc-RSV vector (Invitrogen, Gaithersburg, MD, USA) or HindIII/ BamHI site of pBK-CMV (Stratagene, Cedar Creek, TX, USA) vector. Both pRc-RSV and pBK-CMV vectors contain a geneticin (G418; Mediatech Inc, Herndon, VA, USA) selectable marker. *U94 *DNA sequence in the constructs was confirmed by DNA sequencing. The pBK-U94 construct was specifically used in experiments that necessitated strong expression of U94 protein e.g. immunoblotting, because previous findings showed that *U94 *mRNA and protein were expressed at very low levels [23,24,75]. All plasmid DNA were prepared by double-banded cesium chloride gradient ultracentrifugation. *U94 *construct (pRc-U94 or pBK-U94), or plasmid vector cassette (pRc-RSV or pBK-CMV) was used for transfection of PC3 cells. For transfections, 5.5 × 10^5 ^PC3 cells were plated in 60 mm culture dish, and grown over-night (50%-70% confluence). Transfection was performed by the calcium phosphate-based ProFection Mammalian Transfection method (Promega, Madison, WI, USA) in accordance with manufacturer's protocol. Stably transfected PC3 cells were selected with G418 (600 μg/ml), and expanded to establish U94 recombinant PC3 cell line. Clonal selection was performed on G418 resistant healthy colonies using a clonal cylinder. In order to minimise culture driven genetic changes [76], transfected cells were discarded after 8 passages.

### Protein extraction, and immunoblot analysis

Nuclear fraction from cellular extract was prepared as described previously [77]. Confluent PC3 cell line (10^7^-1.5 × 10^7 ^cells), stably expressing U94 and resistant to G418 antibiotic (Cellgro, Herndon, VA, USA) was freshly prepared by trypsinization, washed with DMEM (Cellgro/Mediatech, VA, USA), suspended in 50 ml DMEM (in sterile 50 ml centrifuge tube), and incubated at 37°C/ 5% CO_2 _for 2 hours. Cells were centrifuged at 200 × g for 5 minutes, and resuspended in 0.5 ml phosphate buffered saline (PBS, Biofluids, Rockville, MD, USA) in microfuge tube. The cell pellet from another round of centrifugation was resuspended in 400 μl of Buffer A (10 mM HEPES; 10 mM KCl; 0.1 mM EDTA; 0.1 mM EGTA; 1 mM DTT; o.5 mM PMSF; 1% v/v aprotinin). After incubation at 40°C for 15 minutes, cells were lysed by adding 0.6% Nonidet P-40, mixed by inverting tube 10 times, and the nuclei was obtained by centrifugation at 200 × g for 5 minutes. The supernatant was used as the cytoplasmic fraction. The nuclei were resuspended gently in ice-cold 100 μl of Buffer B (20 mM HEPES; 0.4 M NaCl; 1 mM EDTA; 1 mM DGTA; 1 mM DDT; 1 mM PMSF; 1% v/v aprotinin; 10% glycerol), using a wide bore pipette. The nuclei lysate was incubated in a rotary shaker for 30 minutes at 4°C, and then centrifuged at 12000 × g for 10 minutes. To aliquots of the clear supernatant in microfuge tubes, 0.025 mg/ml leupeptin was added before storage at -80°C. The control cell lines: vector transfected and resistant to G418, and parental PC3, were similarly treated.

Protein determination in the extracts was performed using BCA protein assay kit (Pierce, Rockford, IL, USA). Following sodium dodecyl sulphate-polyacrylamide gel electrophoresis (SDS-PAGE), resolved proteins were electroblotted onto polyvinylidene difluoride (PVDF) membrane. The membrane was blocked in 5% non-fat milk solution on a rocker for 30 min, and rinsed quickly in Tris/sodium chloride/EDTA/Tween 20 (TNET, 0.2 M Tris pH 7.5; 0.05 M EDTA; 1.0 M NaCl; 1% Tween 20) wash solution. Then the membrane was washed twice in TNET on a rocker for 10 minutes, before it was probed with U94 primary antibody AB679 (Rabbit antiserum, 1:1000 dilution in TNET; Amersham) on a rocker for 1 hour. This was followed by three 10-minutes washes in TNET before anti rabbit-HRP-tagged secondary antibody (1:10,000 dilution in TNET; Amersham) was added and incubated for 1 hour. Three 10-minutes washes in TNET were performed on a rocker, before the detection of immunoreactive proteins using ECL (Amersham, England) reagent.

### Focus formation assay

Two clones of *U94 *transfected cell lines were used for studies. Vector transfected and parental PC3 cell lines were used as controls. Cells were plated at 1 × 10^6 ^cells/ 75 cm^2 ^culture flask in duplicate and grown to confluence. Focus formation was visually detected by observing dense foci of intensive cell growth, consisting of refractive cells that rounded up and piled on top of each other [49]. The number of foci in each flask was noted on the 10th day after the cells were confluent. Average counts of foci in duplicate flasks were determined for each cell type.

### Tumorigenicity assay

The tumorigenicity of PC3 cell line stably expressing U94 was tested in athymic Ncr *nu/nu *mice. The control animals were treated with vector transfected PC3 cell line. In all cases 5 × 10^6 ^cells were inoculated subcutaneously behind the neck, into athymic nude mice as earlier described [78]. The mice were monitored every 2 or 3 days for the appearance of tumors, and tumor volume was measured on days 7, 14, 17, 21 and 28 post inoculation. Tumor sizes were evaluated by tumor volume (length × width × height, in cm). In all cases confluent cells were used. There were two animal experiments. In the first (n = 3 per group), data entries were made on days 0, 7, 17, 21, and 28, while in the second (n = 4 per group) entries were made on days 0, 7, 14, and 17 post inoculation. Data were pooled from the two experiments and reported. The Animal Welfare Committee, Georgetown University, approved the protocol for the animal studies.

### Total RNA extraction and purification

Two clones of PC3 cell line stably expressing U94 (test samples 1 and 2) and PC3 cell line transfected with vector cassette (reference sample) were used for studies. Cells were grown to confluence and total RNA was extracted using Trizol reagent (Invitrogen, Gaithersburg, MD) following manufacturer's instructions. The RNA was cleaned up using the RNeasy^® ^mini columns (Qiagen, Valencia, CA, USA) following manufacturers' instructions. RNA content and quality was initially determined by OD_260_and OD_280 _measurements. RNA samples showing an OD_260/280 _ratio higher than 1.8 was used for microarray hybridization and QRT-PCR. RNA content and integrity was reassayed in duplicate using the Bioanalyzer 2100 (Agilent, Germantown, MD, USA).

### Microarray analysis

Gene expression analysis was performed using a 6 k human cDNA microarray fabricated with ~6000 cancer related genes. Fifty micrograms of total RNA from test and reference samples were separately reversed transcribed using the MicroMax™ Direct cDNA Labeling Kit (Perkin Elmer Life Sciences, Boston, MA, USA) into Cy3 and Cy5 labeled cDNA targets. Cy3-labeled targets prepared from test samples 1 and 2 were hybridized with Cy5-labeled cDNA targets from reference sample onto separate microarrays. A dye-swapping experiment was performed with cDNA targets from test sample 1 labeled with Cy5 and cDNA targets from reference sample labeled with Cy3 in order to eliminate any experimental bias owing to the differences in incorporation efficiency of the 2 fluorescent dyes. Thus the microarray hybridization was carried out in triplicate. Labeled test and reference cDNAs were pooled and purified using Microcon YM-100 filter units (Millipore Corp., Bedford, MA, USA), and co-hybridized onto the microarray at 65°C for 14 hours in the dark. Each microarray was washed at room temperature in 45 ml of the respective wash buffer with the following composition and for the specified duration: 1x SSC/ 0.2% SDS for 5 minutes; 0.5x SSC/ 0.01% SDS for 15 minutes; 0.06x SSC/ 0.01% SDS for 15 minutes; 0.06x SSC for 15 minutes. The washed microarrays were spun at 1000 rpm for 4 minutes before scanning at 5 micron resolution using the ScanArray 5000XL (Packard Biosciences, Billerica, MA, USA). Signals generated from Cy3 and Cy5 channels on each microarray were background subtracted and normalized to the total signals of all spots by LOWESS method, and analyzed by ScanArray Express software (Perkin Elmer Life Sciences, Boston, MA, USA). Data were represented as a fold change of fluorescence intensity of a gene from test sample versus reference sample. A fluorescence intensity ratio of *U94*/vector transfected targets ≥ 2 represented up-regulation of gene; while ≤ 0.5 represented down-regulation. Average values and standard deviation for triplicate experiments were determined. Genes were considered to be differentially expressed only if they displayed the same trend of change in expression in each of the triplicate experiments. Gene annotation information was based on the human Unigene Cluster Build #161 (5th of June 2003; NCBI)

### Quantitative real-time polymerase chain reaction (QRT-PCR)

To verify the expression pattern of the differentially expressing genes identified from microarray experiments, QRT-PCR was performed as described previously (79). Equal amounts of total RNA from test and reference cell lines were treated with DNase 1 (Invitrogen, Gaithersburg, MD, USA), and reverse transcribed using random hexamers and SuperScript II (Invitrogen, Gaithersburg, MD, USA) to prepare the first strand cDNA samples for QRT-PCR analyses. The RT product was diluted 5-fold, and 1 μl is equivalent to 1x concentration. Gene specific primers (Table 3) were designed by Primer Express Version 2.0 (Applied Biosystems, Foster City, CA, USA) according to the sequence information provided for the cDNAs on the microarray. The primers were BLASTed against the non-redundant and EST mouse sets from NCBI to confirm specificity. QRT-PCR was performed in triplicate using SYBR^® ^Green I chemistry on 7900 HTS Sequence Detection System (Applied Biosystems, Foster City, CA, USA) according to manufacturer's instructions. The temperature cycle for QRT-PCR was set up as following: 50°C for 2 minutes; 95°C for 10 minutes; 95°C 15 seconds and 60°C for 1 minute for 40 cycles. A final dissociation cycle running at 95°C for 15 seconds, 60°C for 15 seconds and 95°C for 15 seconds was set up for monitoring the specificity of amplification. The relative standard curve method was used for quantifying gene expression level, in which the C_T _values of a series of fixed amounts of test sample (or reference sample) cDNAs (0.01x , 0.1x and 1x as described above) were plotted against these amounts of cDNAs. The C_T _value for a gene at 0.1x concentration in the reference sample (or test sample) was fitted onto the standard curve to obtain the respective expression level. A smaller C_T _value indicates a higher expression level, and vice versa. Genes showing C_T _values ≥ 40 were considered to be non-expressing. The final gene expression data were reported after normalising to that of 18S RNA.

**Table 3:** Sequences of Forward and Reverse primers for gene amplification in QRT-PCR.

**Genes**	**Forward Primer**	**Reverse primer**
*FN 1*	5-GTGTGACCCTCATGAGGCAAC-3	5-CTGGCCTCCAAAGCATGTG-3
*SERPINE 2*	5-CACATCAGCACCAAGACCATAGAC-3	5-TGCCAAGAACTTTCAGCGG-3
*ADAMST 1*	5-CCAGCGTATCTTGCCAGTAACC-3	5-TTTGCAACTGGCAGTTTACTCTG-3
*ANGPTL 4*	5-CCACTTGGGACCAGGATCAC-3	5-CGGAAGTACTGGCCGTTGAG-3
*SPUVE 23*	5-CCCAGTCTACCCTCAATTTAGCC-3	5-GCAGTGGAGTTCCCTTATGACAC-3

Gene specific primers for QRT-PCR were designed using Primer Express Version 2.0 (Applied Biosystems, Foster City, CA) according to the sequence information provided for the cDNAs on the microarray.

### Statistical analysis

A repeated measures analysis of variance (ANOVA), supplemented by Paired Student's t-test, was used to evaluate the differences in tumor volume between U94 treated and control vector treated mice. A value of p < 0.05 was considered statistically significant. SAS software (v8.2, SAS Institute, Cary, NC, USA) was used for ANOVA. Experimental data, where applicable, are represented as mean ± SD.

## Authors' contributions

ETI performed Cell culture, molecular biology studies, immunoassays, participated in statistical analysis, and drafted the manuscript; ALYP carried out the microarray hybridization and data analysis; WJ performed the QRT-PCR analysis; SM carried out the tumorigenicity studies in mice; WYC performed RNA extraction, data analysis and participated in coordination of the studies; KC and SZ assisted in some of the molecular biology studies, and JC participated in coordination of the studies; LJR conceived of the study, and coordinated the studies. All authors read and approved the final manuscript.

## Competing interests

The author(s) declare that they have no competing interests.
